# Antegrade stenting using a new covered multi-hole metal stent for malignant biliary obstruction in surgically altered anatomy

**DOI:** 10.1055/a-2233-2843

**Published:** 2024-02-02

**Authors:** Kojiro Tanoue, Hirotsugu Maruyama, Yoshinori Shimamoto, Tatsuya Kurokawa, Yuki Ishikawa-Kakiya, Akira Higashimori, Yasuhiro Fujiwara

**Affiliations:** 1 12936 Graduate School of Medicine, Department of Gastroenterology, Osaka Metropolitan University, Osaka, Japan

Endoscopic ultrasound-guided hepaticogastrostomy (EUS-HGS) with antegrade stenting has recently been applied to malignant biliary obstruction including in surgically altered anatomy 1 2 . This method has reduced the rate of bile leakage, which is expected to prevent stent migration 3 , and prolong time to stent dysfunction 4 . In surgically altered anatomy, there is a concern that a covered self-expandable metal stent (SEMS) can cause the other hepatic ducts to occlude as the distance between the anastomosis and bifurcation is small. The commonly used uncovered SEMS may result in tumor ingrowth. We report successful EUS-HGS with antegrade stenting using a new fully covered multi-hole SEMS (HANARO Biliary Multi-Hole NEO; M.I.Tech Co Ltd., Pyeongtaek, South Korea) ( Fig. 1 ) for malignant biliary obstruction in surgically altered anatomy.

**Fig. 1:**
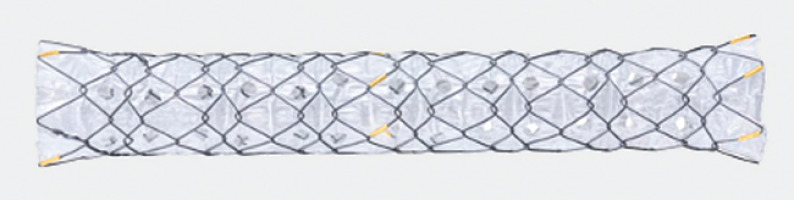
New fully covered multi-hole metal stent. This stent is made from nitinol with a fixed hook and cross-wired structure, which is fully covered with a silicone membrane and a multi-hole of 1.8 mm in diameter.

A 51-year-old man, who previously underwent subtotal stomach-preserving pancreatoduodenectomy for distal cholangiocarcinoma (pT2N1M0 pStage IIB) and subsequent chemotherapy, visited our department for obstructive jaundice. He was diagnosed with a tumor recurrence through computed tomography ( Fig. 2 ) and endoscopic ultrasound ( Fig. 3 ). The procedure involved a B3 puncture using a 22-gauge needle and 0.018-inch guidewire, fistula dilation, and catheter insertion followed by the placement of two guidewires. Fluoroscopy showed that the left and right hepatic ducts in the bifurcation were not separated. Thereafter, the two guidewires and catheter were successfully advanced to the jejunum over the anastomosis, and we confirmed the anastomotic obstruction. Finally, an 8-mm fully covered multi-hole SEMS was placed from the jejunum into the left hepatic duct followed by the placement of a 7 Fr plastic stent into the EUS-HGS fistula ( Video 1 ). After the procedure, obstructive jaundice improved. The placement of a covered SEMS for malignant biliary obstruction in surgically altered anatomy is usually difficult to position and requires caution. However, antegrade stenting using this stent is an appropriate indication of malignant biliary obstruction and can be successfully performed without hesitation.

**Fig. 2:**
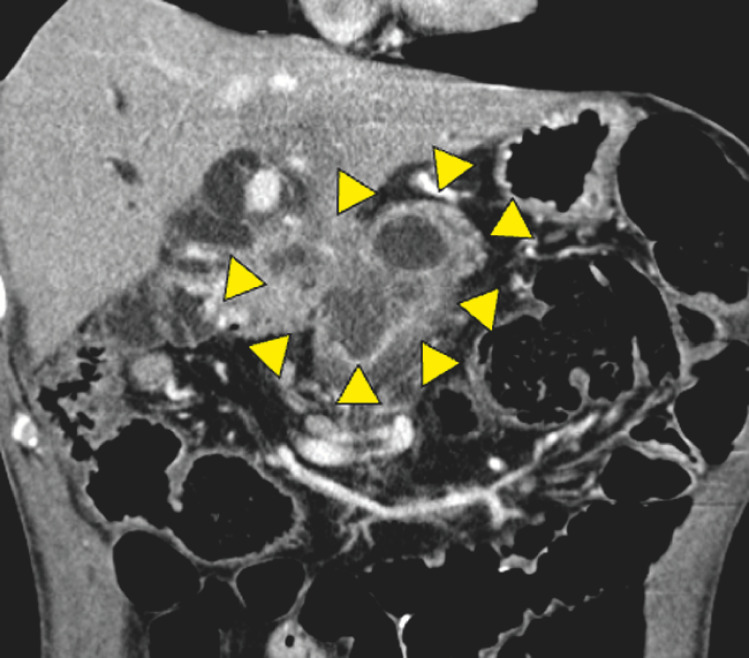
Malignant hilar biliary obstruction by recurrence of cholangiocarcinoma as visualized by computed tomography examination.

**Fig. 3:**
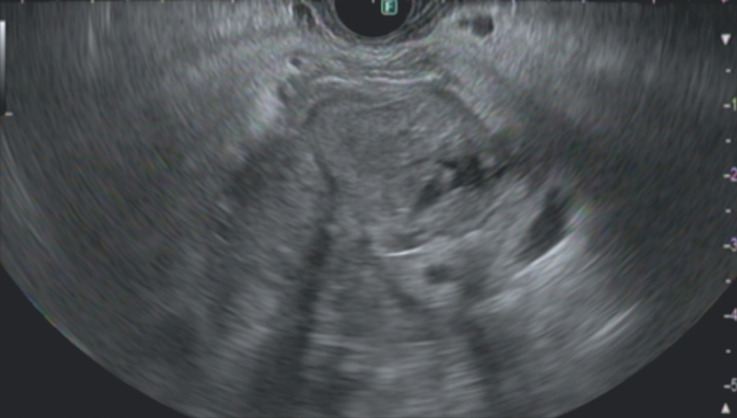
Malignant hilar biliary obstruction by recurrence of cholangiocarcinoma as visualized by endoscopic ultrasonography.

Antegrade stenting using a new covered multi-hole metal stent for malignant biliary obstruction in surgically altered anatomy.Video 1

Endoscopy_UCTN_Code_TTT_1AS_2AD

## Notes

Endoscopy E-Videos https://eref.thieme.de/e-videos
*E-Videos* is an open access online section of the journal *Endoscopy* , reporting on interesting cases and new techniques in gastroenterological endoscopy. All papers include a high-quality video and are published with a Creative Commons CC-BY license. Endoscopy E-Videos qualify for HINARI discounts and waivers and eligibility is automatically checked during the submission process. We grant 100% waivers to articles whose corresponding authors are based in Group A countries and 50% waivers to those who are based in Group B countries as classified by Research4Life (see: https://www.research4life.org/access/eligibility/ ). This section has its own submission website at https://mc.manuscriptcentral.com/e-videos .
